# Exploratory and confirmatory factor analysis of the sensitivity to punishment and sensitivity to reward questionnaire-revised and its psychometric evaluation among Chinese person with substance use disorder

**DOI:** 10.3389/fpsyg.2024.1351450

**Published:** 2024-06-12

**Authors:** Shuying Li, Dongdong Xie, Meiting Li, Qingqing Che, Jun Zhang, Xingwei Luo, Taisheng Cai

**Affiliations:** ^1^Medical Psychological Center, Second Xiangya Hospital, Central South University, Changsha, China; ^2^Medical Psychological Institute, Second Xiangya Hospital, Central South University, Changsha, China; ^3^Department of Corrective Education, Hunan Judicial Police Vocational College, Changsha, China

**Keywords:** person with substance use disorder, reinforcement sensitivity theory, SPSRQ, measurement invariance, gender difference

## Abstract

The Sensitivity to Punishment and Sensitivity to Reward Questionnaire (SPSRQ) is a self-report tool widely used to assess individuals' level of reinforcement sensitivity. Drug addiction is strongly associated with reinforcement sensitivity, but there is a lack of measurement tools to assess reinforcement sensitivity in drug users, necessitating the revision and application of the SPSRQ among drug users. This study recruited 819 drug users (mean age = 34.74; 56.41% female) from five compulsory rehabilitation centers in Hunan Province, China. The applicability of the SPSRQ among person with substance use disorder was assessed by conducting reliability analyses and validity analyses, with retesting performed by 127 individuals after 6 weeks. Exploratory factor analysis for the SPSRQ showed a stable two-factor structure in person with substance use disorder. Confirmatory factor analysis indicated acceptable goodness of fit indexes for the two-factor structure. The SPSRQ also demonstrated good reliability and convergent and discriminant validity evidence. The two-factor structure of the SPSRQ also demonstrated measurement invariance across gender. Further comparative analysis found that the degree of reward sensitivity was higher for males than for females. Generally, the SPSRQ has shown evidence of good reliability and validity in Chinese drug-dependent populations, and it is suitable for research and application with Chinese person with substance use disorder. These findings about the personality traits of people with substance use disorder provide a solid basis for further research.

## 1 Introduction

Drug use and abuse has long been a serious challenge worldwide. In 2021, more than 296 million people worldwide reported using drugs, with 39.5 million people suffering from drug use disorders, a prevalence which has increased by 45% over the past decade (United Nations Office on Drugs Crime, 2023). Drug use can not only seriously endanger the physical and mental health of individuals and increase their risk of developing mental illnesses and substance use disorders, but it can also threaten social security and stability. According to the neuroadaptive and behavioral models of drug dependence, there is an interaction between the neurophysiological and behavioral processes of drug dependence, and exploring the psychobehavioural processes behind dependence is important for the development of therapeutic interventions (Degenhardt et al., 2010). The reinforcement sensitivity of person with substance use disorder has therefore been receiving increasing attention in particular.

Reinforcement sensitivity refers to an individual's reactivity in their emotional, motivational, and behavioral systems when presented with a reinforcer (Gray, 1970). Reinforcement sensitivity theory suggests that reinforcement sensitivity consists of two systems: the behavioral inhibition system (BIS), which reflects one's tendency to respond to negative stimuli and corresponds to punishment sensitivity, and the behavioral activation system (BAS) which is one's tendency to respond to positive stimuli, and corresponds to reward sensitivity (Smillie and Jackson, 2006). Reinforcement sensitivity is an important personality trait strongly associated with drug abuse, with research findings supporting the association between the two. Compared to non-user controls, people who are dependent on methamphetamine-type drugs are more sensitive in both their BIS and BAS (Alemikhah et al., 2016). Reinforcement sensitivity is an important behavioral mechanism in the context of drug abuse. An in-depth exploration of the characteristics of reinforcement sensitivity in drug treatment populations may provide insight into the behavioral reward and punishment mechanisms of drug abuse and facilitate drug treatment programs and interventions.

However, there is a lack of measurement tools which can be used to assess the reinforcement sensitivity of person with substance use disorder, and therefore a need to revise and the reinforcement sensitivity scale for application to person with substance use disorder. In practice, the most widely-used self-assessment scales are the Behavioral Inhibition/Behavioral Activation System Scale (BIS/BAS) and the Sensitivity to Punishment and Sensitivity to Reward Questionnaire (SPSRQ). Compared to the BIS/BAS scale, which is controversial in terms of its factor structure, the SPSRQ is considered to be more stable in terms of its factor structure, and because it explicitly contains both punishment sensitivity and reward sensitivity factors, which is consistent with reinforcement sensitivity theory (Torrubia et al., 2001). Moreover, the stability of the two-factor structure of the SPSRQ has been validated in empirical studies (Aluja and Blanch, 2011; Dufey et al., 2011; Conner et al., 2018) (see Table 1). In fact, Conner et al. (2018) tested the SPSRQ-Revised and Clarified (SPSRQ-RC) and found that it has two one-dimensional factors, which is consistent with the two-factor structure of reinforcement sensitivity theory, and their findings suggest that the SPSRQ-RC has good psychometric properties.

**Table 1:** Goodness-of-fit indexes of the SPSRQ.

**Author**	***χ^2^***	***df***	***p***	***CFI***	***RMSEA***
Aluja and Blanch (2011)	423.44	169	< 0.001	0.89	0.040
Dufey et al. (2011)	1950.87	859	0.001	0.68	0.053
Conner et al. (2018)	933.42	167	< 0.001	/	0.067

χ^2^= chi-square; df, degree of freedom; CFI, comparative fit index; RMSEA, Root mean square error of approximation; SPSRQ, The Sensitivity to Punishment and Sensitivity to Reward Questionnaire.

Although the factor structure of the SPSRQ has been widely validated, different cultures report differences in the results of the revised versions of the SPSRQ. While the SPSRQ was first developed by Torrubia et al. (2001), it has since been translated into several languages and revised several times. Some studies have retained the 48 items of the original scale, for example, Li et al. (2007) applied the Chinese version of the SPSRQ as translated by Torrubia to university students in Taiwan. However, most studies and revisions have deleted items which exhibited lower factor loadings, with different cultures adopting versions of the scale, such as 44 (Chile), 35 (France), and 20 (Spain) questions (Lardi et al., 2008; Aluja and Blanch, 2011; Dufey et al., 2011). In addition, sample groups used in most studies have tended to focus on the college student population. Therefore, the present study aimed to develop a revised SPSRQ for use in the Chinese drug-dependent population.

Impulsivity is closely associated with substance use problems. It is associated with negative behaviors and negative affect such as imprudence (Claes et al., 2000). Impulsive behaviors are present in patients with cannabis use disorders, with the prevalence of high levels of impulsivity ranging from 30% to 33% (Wagner et al., 2022). Meanwhile, Richardson et al. (2014) found that people who are more sensitive to rewards and threats may be the most impulsive and more susceptible to substance use. Based on the above studies, the BIS/BAS self-report scale measures reinforcement sensitivity, the present study used the BIA/BAS to evaluate the convergent validity of the SPSRQ. In addition, drug abuse and impulsivity are uniquely associated with reinforcement sensitivity, so the Drug Dependence Scale [SDDS; (Li et al., 2021)] and the Impulsive Behavior Scale [S-UPPS-P; (Cyders et al., 2014)] were chosen to test the discriminant validity of the SPSRQ.

Previous research has suggested that gender differences may exist in punishment sensitivity and reward sensitivity (Torrubia et al., 2001). Li et al. (2007) found that males showed greater reward sensitivity than females among Taiwanese college students. A prerequisite for comparing group gender differences in scale results is determining the measurement invariance of applying the scale to both males and females, otherwise it is not possible to determine whether the between-group differences are due to inequalities in the scales themselves, or whether males and females in fact differ (Meredith and Teresi, 2006). Therefore, the current study tested the SPSRQ in a sample comprising both males and females to determine the punishment sensitivity and reward sensitivity according to gender.

In summary, the purpose of the present study was to investigate the applicability of the SPSRQ in a Chinese, drug-dependent population. After revising the SPSRQ for relevance in this specific demographic, we assessed the reliability, internal structure validity, convergent and discriminant validity evidence of the scale, and tested whether the SPSRQ had measurement invariance in both male and female drug-dependent populations.

## 2 Materials and methods

### 2.1 Participants and procedures

The present study collected two samples (i.e., Sample 1 and Sample 2) from June to July 2018 and from July to August 2019 at five compulsory isolation drug rehabilitation centers in Hunan Province, China. The inclusion criteria for participants were as follows: (i) at least 18 years old; (ii) met the diagnostic criteria for substance use disorders according to the Diagnostic and Statistical Manual for Mental Disorders, Fifth Edition [DSM-5; (American Psychiatric Association, 2013)]; (iii) had already completed the acute-phase detoxification treatment and had not suffered from severe physical withdrawal symptoms; and (iv) was a voluntarily participant in the study, and was able to read Chinese. The exclusion criteria were: suffering from severe physical illness or from severe mental disorders.

Before administering the test, the researcher explained to the subjects that the results of the questionnaires would not to be used as an indicator for reward or punishment, and would not affect participants' lives in the detoxification center. After ensuring that all subjects understood the contents of the questionnaires, participants began to complete the measures. During this time, detoxification center staffs were not allowed to view subjects' answers and were kept at a distance from the participants. Responses were checked for completeness on the spot. Paper questionnaire data were entered by two people with comparison checks. A total of 895 participants completed the questionnaires and 819 participants were valid, resulting in an effective recovery rate of 91.51%. The average length of time they had substance use disorder was 89.37 months; 78.27% of them used multiple substances (Cannabis, Hallucinogens, Opioids, *etc*.); the average length of time they were substance free was 10.72 months. The basic demographics of Sample 1 and Sample 2 are shown in Table 2.

**Table 2:** Basic demographic data of sample 1 and sample 2.

	**Sample 1 (*n* = 429)**	**Sample 2 (*n* = 390)**
Age, years	35.10 (SD = 8.22)	34.34 (SD = 7.60)
Female, *n* (%)	175 (40.79)	287 (73.59)
**Education level**, ***n*** **(%)**		
Primary school and below	74 (17.25)	64 (16.41)
Junior high school	213 (49.65)	192 (49.23)
High school and above	142 (33.10)	134 (34.36)
**Working status**, ***n*** **(%)**		
Employed	177 (41.26)	180 (46.15)
Unemployed	185 (43.12)	165 (42.31)
Other status	67 (15.62)	45 (11.54)
**Marital status**, ***n*** **(%)**		
Unmarried	147 (34.27)	109 (27.95)
Married	106 (24.71)	93 (23.85)
Divorced	131 (30.54)	121 (30.77)
Other status	45 (10.49)	67 (17.18)

Sample 1 included person with substance use disorder from four compulsory isolation and drug rehabilitation centers. A total of 429 valid SPSRQ responses were collected for item analysis, exploratory factor analysis, and reliability analysis. Six weeks later, 130 subjects were randomly selected from Sample 1 using coded numbers. These codes were used to generate random numbers, identifying and matching SPSRQ results at a later date. 127 valid questionnaire responses were collected for retest reliability analysis. The mean age of participants in Sample 1 was 35.10 years (SD = 8.22, ranging from 18 to 57 years of age), and the proportion of females was 40.79% (*n* = 175).

Sample 2 consisting of 390 participants from one other compulsory isolation and drug rehabilitation center, completed the SPSRQ, the BIS/BAS, the S-UPPS-S and the SDDS. It was used for confirmatory factor analysis, convergent and discriminant validity analysis, and reliability analysis. The mean age of participants in Sample 2 was 34.34 years (SD = 7.60, ranging from 18 to 54 years of age), and 73.59% were female (*n* = 287).

All participants had volunteered to take part in the present study, and provided their informed consent before the study commenced. This study was approved by the Ethics Committee of the Second Xiangya Hospital of Central South University in China.

### 2.2 Measures

#### 2.2.1 The sensitivity to punishment and sensitivity to reward questionnaire

The SPSRQ was developed by Torrubia et al. (2001) to measure individuals' behavioral tendencies toward punishment and reward stimuli. The SPSRQ has a total of 48 items measuring two subscales: punishment sensitivity and reward sensitivity. Each item is scored as either 0, indicating no, or 1, indicating yes. The higher the total score, the stronger the individual's sensitivity to punishment or reward. Permission for the use and revision of this scale in this study was obtained from the original scale creator. The research team translated the SPSRQ into Chinese Mandarin following the usual procedure for scale translation in cross-cultural research. Consideration was given to specific expression habits and characteristics in the Chinese context, after discussion, the Chinese version of the scale was modified to create the Chinese version of the SPSRQ.

#### 2.2.2 The behavioral inhibition/behavioral activation scales

The BIS/BAS was developed by Carver and White (1994) to assess an individual's level of reinforcement sensitivity. The 24-item scale is divided into two parts, one measuring the Behavioral Inhibition System (BIS), which relates to anxiety and fear factors, and the other to the Behavioral Activation System (BAS), which relates to drive, reward responsiveness, and fun-seeking factors. Each item is rated on a four-point scale, with 1 being “very much in line” and 4 being “very much out of line.” After taking reverse scoring into account, the higher the total score, the higher the individual's level of behavioral inhibition or behavioral activation. The Chinese version of the scale has been shown to have good reliability (Che et al., 2020). In this study, the Cronbach's alpha of the scale in Sample 2 was 0.807.

#### 2.2.3 The short version of the UPPS-P impulsive behavior scale

The S-UPPS-P was developed by Cyders et al. (2014) to measure an individual's level of behavioral impulsivity. It consists of 20 items, each scored on a four-point scale with 1 being “very non-compliant” and 4 being “very compliant.” Higher scores indicate higher levels of impulsivity. The scale has been shown to have good reliability and validity in Chinese samples (Che et al., 2020). In the current study, the Cronbach's α of the total scale in Sample 2 was 0.775.

#### 2.2.4 The synthetic drug dependence scale

The SDDS was developed by Li et al. (2021) to measure drug dependence among drug users. It consists of 11 items with each scored on a four-point scale, and the severity of drug dependence is evaluated by summarizing items scores, with higher total scores indicating a greater degree of drug dependence. The scale has been shown to have good reliability and validity among Chinese person with substance use disorder (Li et al., 2021). In the current study, the Cronbach's α of the total SDDS scale in Sample 2 was 0.801.

### 2.3 Data analysis

SPSS 26.0 and Mplus 8.3 were used for data processing and analysis. Item analysis, exploratory factor analysis (EFA), and reliability analysis were conducted for Sample 1. Confirmatory factor analysis (CFA), convergent and discriminant validity analysis, and reliability analysis were conducted on Sample 2. Measurement invariance of the SPSRQ was tested according to gender and comparison of differences in punishment vs. reward sensitivity across the total sample (i.e., Sample 1 and Sample 2, *n* = 819).

Item analyses were carried out using the item-total correlation coefficient method and the high-low group test for Sample 1. Corrected item-to-total correlation (CITC) was calculated for each item as well as for the total score, and if the CITC value was over 0.30 and positively correlated, the item was retained; conversely, if the item value was below 0.30, it was considered for deletion. Subjects were divided into a high or low group based on the 27% before and after total SPSRQ score, and an independent samples *t*-test was used to calculate the difference between the two groups for each item score. When the difference was significant (*p* < 0.05), it indicated that the item had good discriminatory power and should be retained; if it was not significant, combined the concept correlation of specific items in the Chinese translated scale with their consistency with foundational theories to eliminate items.

The internal structure validity evidence of the scale was then assessed using EFA and CFA. EFA, CFA and measurement invariance analyses were performed using Mplus 8.3 software. In EFA, two SPSRQ factors were extracted using the weighted least squares mean and variance adjusted (WLSMV); if the factor loading was higher than 0.40, the item was retained, and if not, the item was considered for deletion. In this study, the internal structure validity evidence of the scale was examined using CFA. WLSMV is an analytical method specifically designed to deal with categorical variables in the application of the categorical variables with five or fewer options, resulting in fewer standard errors and more accurate parameter estimates (Bandalos, 2014). As the SPSRQ has a binary variable, the WLSMV method was chosen in this study as the CFA estimation method. When evaluating goodness of fit indexes, different types of fitness indicators, factor loadings, and theoretical foundations should be combined before making a comprehensive judgement (Byrne, 1998). Therefore, to assess goodness of fit indexes, the ratio of chi-square to degrees of freedom (χ^2^/*df*), root mean square error of approximation (*RMSEA*), comparative fit index (*CFI*), and Tucker-Lewis index (*TLI*) were used as indicators. An acceptable model should meet the following criteria: the value of χ^2^/*df* in the range of 1 to 3 (Browne and Cudeck, 1992), the *RMSEA* value in the range of 0.05 to 0.08, the upper limit of the 90% *RMSEA* confidence interval (*CI*) being < 0.10 (Kline, 2015), and the values of *CFI* and *TLI* are > 0.90 (Bentler and Bonett, 1980).

Measurement invariance of the SPSRQ in terms of gender was tested in the final model. Measurement invariance analysis is usually divided into three steps: (i) configural invariance, which tests whether the latent variable composition of the scale is the same across groups; (ii) metric invariance, which tests whether the factor loadings of the entries are equal across all groups; and (iii) scalar invariance, which tests whether the thresholds of the observed variables are equal across all groups. Whereas, the WLSMV method was chosen due to the binary variables in this study, as it allows scale factors or residual variances to vary across groups, it does not allow for the recognition of unit invariance, so only configural invariance and scalar invariance were tested (Muthén and Muthén, 2017). When comparing the morphological invariance model to the scale invariance model, the measurement invariance model was considered to be acceptable when Δ*CFI* < 0.010 and Δ*RMSEA* < 0.010 (Chen, 2007). After measurement invariance was established, independent sample *t*-tests were used to test for gender differences in reinforcement sensitivity among person with substance use disorder.

The internal consistency reliability of the SPSRQ was rated by calculating the Cronbach's α and composite reliability for each dimension of the SPSRQ as well as for the total scale. The composite reliability is calculated by the standardized factor loading value of each item. If the value is > 0.70, it indicates that each item has good consistency in content (Nunnally, 1978). Correlation analyses were conducted on the total scale scores of 127 subjects selected randomly from Sample 1 for the pre- and post-tests, and the intraclass correlation coefficients of the two scores were used as an indicator of test-retest reliability. In addition, the Spearman correlation coefficients of the relationships between the SPSRQ scores and the scores of the three related scales (i.e., BIS/BAS, SDDS, and S-UPPS-P) were used as indicators for assessing the convergent and discriminant validity of the SPSRQ. Then use the *cocor* installation package of R language programming software to test the statistical differences between the correlation coefficients (Olivier et al., 2015).

## 3 Results

### 3.1 Item analyses

Item analyses of the 429 valid responses from Sample 1 showed that, in testing the differences between the high and low subgroups, the differences between the scores of the high and low subgroups were statistically significant for all items (*p* < 0.01). Furthermore, most of the items had values > 0.30 for the corrected item-to-total correlation (CITC), while items 1, 3, 4, 7, 8, 11, 17, 25, 27, 28, 29, 32, 34, 36, and 40 all had CITC values lower than 0.30. After deleting the 15 abovementioned items, the remaining 33 items were recalculated and the results showed that the CITC values for items 2, 9, 26, and 42 were all below 0.30. The deletion of these four items resulted in 29 items remaining (see Table 3).

**Table 3:** Sample 1 SPSRQ item analyses (*n* = 429).

**Item**	**SP**			**Item**	**SR**		
	**Discrimination-*t***	**CITC**	**Cronbach's α if item deleted**		**Discrimination-*t***	**CITC**	**Cronbach's α if item deleted**
5	10.259^***^	0.462	0.801	6	9.398^***^	0.360	0.779
13	8.645^***^	0.366	0.807	10	8.868^***^	0.422	0.774
15	5.928^***^	0.357	0.808	12	7.554^***^	0.516	0.766
19	8.645^***^	0.494	0.798	14	9.080^***^	0.398	0.776
21	7.382^***^	0.343	0.809	16	9.793^***^	0.371	0.779
23	8.961^***^	0.367	0.807	18	8.876^***^	0.481	0.769
31	10.407^***^	0.493	0.798	20	6.350^***^	0.474	0.770
33	10.714^***^	0.429	0.803	22	6.924^***^	0.370	0.779
35	7.254^***^	0.460	0.801	24	6.081^***^	0.340	0.781
37	12.195^***^	0.400	0.805	30	6.277^***^	0.394	0.777
39	12.017^***^	0.435	0.803	38	13.927^***^	0.303	0.784
41	12.960^***^	0.522	0.796	44	3.184^***^	0.384	0.777
43	12.643^***^	0.436	0.803	46	7.347^***^	0.468	0.770
45	9.035^***^	0.341	0.809	48	6.007^***^	0.382	0.778
47	10.114^***^	0.486	0.799				

^***^p < 0.001; SP, sensitivity to punishment; SR, sensitivity to reward; CITC, corrected item-to-total correlation.

### 3.2 Internal structure validity

EFA of the remaining 29 items using data from Sample 1 (*n* = 429) showed a Kaiser-Meyer-Olkin value of 0.861 (> 0.70) and a Bartlett's test of sphericity value of 2493.015, *df* = 406, *p* < 0.001, indicating that the data was suitable for factor analysis. Extracting two factors using WLSMV, the cross-loading of item 16 was 0.355 (> 0.30), and the difference between the factor loadings and the cross loadings both of items 6 and 16 were < 0.2; item 38 had a factor loading of 0.368 (< 0.40) on the reward sensitivity factor, a cross-loading of 0.490 (> 0.30), and the difference between the two was < 0.2. After deleting items 6, 16 and 38, EFA was conducted once again, and the results showed that the difference between the factor loadings and the cross loadings of item 45 was < 0.2. After deleting the four above-mentioned items, EFA was conducted on the remaining 25 items, and all of them had factor loadings >0.40 (see Table 4). Thus, the final version of the SPSRQ containing 25 items was finalized.

**Table 4:** Factor loadings in EFA (Sample 1, *n* = 429) and CFA (Sample 2, *n* = 390).

**Item**	**SP**		**Item**	**SR**	
	**EFA**	**CFA**		**EFA**	**CFA**
5	0.608	0.557	10	0.589	0.621
13	0.508	0.552	12	0.699	0.626
15	0.553	0.611	14	0.524	0.543
19	0.737	0.679	18	0.676	0.683
21	0.496	0.532	20	0.644	0.679
23	0.512	0.508	22	0.507	0.569
31	0.692	0.707	24	0.495	0.518
33	0.587	0.573	30	0.632	0.568
35	0.698	0.569	44	0.659	0.555
37	0.517	0.479	46	0.687	0.667
39	0.552	0.634	48	0.587	0.481
41	0.695	0.636			
43	0.566	0.619			
47	0.703	0.733			

SP, sensitivity to punishment; SR, sensitivity to reward; EFA, exploratory factor analysis; CFA, confirmatory factor analysis.

The results of fitting the SPSRQ two-factor model using Sample 2 (*n* = 390) showed that there were no negative error variances nor any standardized correlation coefficients >1, and that the parameter estimates were all within reasonable ranges, indicating that the model has a good basic fit. Further analysis of the overall goodness of fit indexes showed that: χ^2^/*df* = 1.933, *CFI* = 0.881, *TLI* = 0.870, *RMSEA* = 0.049 [0.043, 0.055], with *CFI* and *TLI* values both lower than 0.90, and factor loadings on all items >0.40, with the factor loadings ranging from 0.479 to 0.733 (see Table 4).

### 3.3 Reliability analysis

In Sample 1 (*n* = 429), the Cronbach's α values of the total SPSRQ scale, punishment sensitivity, and reward sensitivity were 0.804, 0.809, and 0.768, respectively, and their values of composite reliability were 0.933, 0.887, and 0.856, respectively. Test-retest reliability was assessed using the same 127 valid responses from in Sample 1 used previously, and the re-test data at an interval of 6 weeks resulted in intraclass correlation coefficients between the initial and retest total scale scores and the two factors being 0.735–0.752 (*p* < 0.01). In contrast, using responses from Sample 2 (*n* = 390) against the total sample scores (*n* = 819), the Cronbach's αs for the total SPSRQ were 0.791 and 0.798, respectively, the Cronbach's αs for punishment sensitivity were 0.796 and 0.803, respectively, and the Cronbach's αs for reward sensitivity were 0.756 and 0.762, respectively.

### 3.4 Convergent and discriminant validity

The results of the correlation analyses showed that the total scores of the revised 25-item SPSRQ were significantly and positively correlated with the total scores of the BIS/BAS scale, the S-UPPS-P scale, and the SDDS scale (see Table 5). To be specific, the correlation coefficient between the SPSRQ scale and the BIS/BAS scale was 0.451, and the correlation coefficients with the S-UPPS-P scale and the SDDS scale were 0.338 and 0.125. Furthermore, the correlation coefficient between the SPSRQ scale and the BIS/BAS scale was significantly higher than the correlation coefficients with the S-UPPS-P scale (*p* < 0.05) and with the SDDS Scale (*p* < 0.001).

**Table 5:** Spearman correlations between SPSRQ scales and other measures in sample 2 (*n* = 390).

	**SP**	**SR**	**SPSRQ**	**S-UPPS-P**	**SDDS**
BIS	0.366^***^	0.128^***^	0.345^***^	0.082	0.093
BAS-D	0.098	0.441^***^	0.328^***^	0.109^*^	0.087
BAS-R	0.158^**^	0.350^***^	0.322^***^	0.055	0.119^*^
BAS-F	0.241^***^	0.281^***^	0.350^***^	0.387^***^	0.115^*^
BIS/BAS	0.297^***^	0.392^***^	0.451^***^	0.194^***^	0.138^**^
SP		0.165^***^	0.807^***^	0.385^***^	0.137^**^
SR			0.691^***^	0.091	0.051
SPSRQ				0.338^***^	0.125^**^
S-UPPS-P					0.147^**^

^*^p < 0.05, ^**^p < 0.01, ^***^p < 0.001; SP, sensitivity to punishment; SR, sensitivity to reward; SPSRQ, The Sensitivity to Punishment and Sensitivity to Reward Questionnaire; S-UPPS-P, Short Version of the UPPS-P Impulsive Behaviour Scale; SDDS, Synthetic Drug Dependence Scale; BIS, behavioural inhibition system; BAS-D, behavioural activation system–drive; BAS-R, behavioural activation system–reward responsiveness; BAS-F, behavioural activation system–fun-seeking; BIS/BAS, The Behavioral Inhibition System and Behavioural Activation System Scale.

### 3.5 Tests of measurement invariance across gender

Measurement invariance testing for gender was conducted using the total sample (*n* = 819). As shown in Table 6, for gender, the two-factor structure of the SPSRQ had an acceptable goodness of fit indexes in both the configural invariance model and the scalar invariance model. Further comparison of the scalar invariance model with the configural invariance model showed Δ*CFI* < 0.010 and Δ*RMSEA* < 0.010, indicating that the SPSRQ measurement invariance holds true for gender, and that the scale scores can be compared across groups.

**Table 6:** Confirmatory factor analysis of multiple nested model fitting indices (gender invariance test).

**Model**	***χ^2^*(*df*)**	***CFI***	***TLI***	***RMSEA* (90%*CI*)**	**Δ*CFI***	**Δ*RMSEA***
Male	436.869^*^(274)	0.914	0.906	0.041 (0.034, 0.048)		
Female	678.921^*^(274)	0.861	0.848	0.057 (0.051, 0.062)		
Configural	1118.691^*^(548)	0.881	0.869	0.050 (0.046, 0.055)		
Scalar	1179.192^*^(669)	0.872	0.865	0.051 (0.047, 0.055)	−0.009	< 0.01

^*^p < 0.05; χ^2^, chi-square; *df*, degree of freedom; *CFI*, comparative fit index; *TLI*, Tucker-Lewis index; *RMSEA*, Root mean square error of approximation.

### 3.6 Comparison of scale scores of subjects across gender

The results of the independent samples *t*-test showed that male person with substance use disorder scored significantly higher than female person with substance use disorder in both the total SPSRQ score and the reward sensitivity score, but the difference between the two groups in punishment sensitivity scores was not significant (see Table 7).

**Table 7:** Comparison of SPSRQ scores across gender.

	**Male (*n* = 357)**	**Female (*n* = 462)**	***t***
SPSRQ	14.01 (SD = 5.08)	13.21 (SD = 5.00)	2.27^*^
SP	8.05 (SD = 3.56)	7.76 (SD = 3.64)	1.15
SR	5.97 (SD = 2.90)	5.45 (SD = 2,94)	2.50^*^

^*^p < 0.05; SP, sensitivity to punishment; SR, sensitivity to reward; SPSRQ, The Sensitivity to Punishment and Sensitivity to Reward Questionnaire.

## 4 Discussion

In this study, the SPSRQ was revised in Chinese and analyzed for reliability and validity by selecting a sample of males and females attending drug rehabilitation programs in China. With good reliability and validity, as well as cross-cultural applicability and stability, the revised SPSRQ showed good applicability in Chinese drug rehabilitation populations.

The results of the item analyses showed that 19 items had CITC values below the 0.30 criterion, indicating that these items were weakly correlated with the total score. A review of the results of previous studies revealed that these items also performed poorly during the revision processes for several different versions of the SPSRQ. For example, items 1, 8, 11, 29, 32, and 34 all performed poorly in the study of Cogswell et al. (2006), with factor loadings below 0.30; in Cooper and Gomez (2008), items 3, 4, 25, and 28 were excluded because of their small contributions to the overall measurement accuracy of the scale; items 3, 7, 9, 22, 27, 29, 36, 40, and 42 were all excluded in Conner et al. (2018) because of low factor loadings or cross-loadings below 0.30. The detailed process of deleting a specific project based on its semantic content can be found in the Supplementary material.

Among remaining 29 SPSRQ items, the EFA results indicated low factor loading for item 38 and high cross-loadings for items 16 and 38. Following Howard (2016)'s recommendation that factor loadings should be > 0.40, cross-loadings < 0.30, and the difference between loadings of the same item on different factors should be > 0.20, this study deleted the items 6, 16, 38 and 45. For item 38, “Do you sometimes do things for quick gains?” which in Chinese context implies potential failure by neglecting long-term interests, this suggests that the content did not clear align with either punishment or reward sensitivity. After reviewing previous research and the specific content of low-performing items, it was found that the quality issues of most of these 23 items were consistent across different cultures, conflicting with the original measurement goal of the scale, and failing to accurately reflect the intended content. Considering the semantic and professional meanings of these specific items in the Chinese culture context, all 23 items mentioned above were decided to be deleted.

The subsequent CFA showed that the factor loadings of the items and the absolute fit indicators χ^2^*/df* and *RMSEA* met the requirements, but the values of the relative fit indicators *CFI* and *TLI* did not reach the recommended value of 0.90. Previous studies on the original 48-question version of the scale generally showed poor fit with most *CFI* values below 0.75 (O'Connor et al., 2004; Cogswell et al., 2006; Aluja and Blanch, 2011). Although the lower absolute fitness index of the SPSRQ in model fitting is common, the values of χ^2^*/df* and *RMSEA* in the study were at acceptable levels, suggesting the hypothesized model could not be rejected on these indicators. The excessive number of factor-corresponding items in the SPSRQ might explain this phenomenon, as studies have found a negative correlation between model fit and an increased number of items (Anderson and Gerbing, 1984). After removing some of these problematic items, the model fit metrics improved. Aluja and Blanch (2011) revised the SPSRQ to include 20 remaining items, achieving a *CFI* value close to 0.90. Thus, the results of *CFI* and *TLI* in this study align with existing empirical studies, though slightly lower than the usual standard of 0.90, possibly due to the excessive number of items included in the scale; the absolute fit indices and factor loadings are in line with the requirements of acceptable two-factor model fit, however, which indicates that the scale has good internal structure validity. The correlation coefficient between the BIS/BAS scale and the SPSRQ scale was 0.451, reaching a moderate correlation, suggesting consistent results between the SPSRQ scale and different instruments that also measure reinforcement sensitivity. In contrast, the correlation coefficient of the SPSRQ scale with the S-UPPS-P scale, and the SDDS scale was significantly lower than the correlation coefficient of the SPSRQ scale with the BIS/BAS scale, suggesting that the correlation between the SPSRQ scale and the scales that measure different components is weak. This indicates that our revised SPSRQ has good convergent and discriminant validity (Zhu, 2000).

The reliability analyses of the revised SPSRQ showed Cronbach's αs for the total scale and both factors in the total sample were all exceeding 0.70, with the α for the punishment sensitivity factor being slightly higher than for the reward sensitivity factor, consistent with the original scale (Torrubia et al., 2001). Meanwhile, the values of composite reliability for the total SPSRQ scale and its two factors were all above 0.70, and the values of the two internal consistency reliabilities met the recommended requirements, indicating that the entries had good consistency in terms of content. Meanwhile, the test-retest reliability of the total SPSRQ scale and its two factors ranged from 0.735 to 0.752, indicating good stability over time.

This study tested the revised SPSRQ for measurement invariance across gender, confirming the scale does meet the requirements for measurement invariance. Comparative analyses revealed that male person with substance use disorder scored significantly higher than female person with substance use disorder on both the total scale and in reward sensitivity. While males scored higher than females in punishment sensitivity as well, the difference between the two groups was not significant, consistent with Li et al. (2007). This suggests that, in the Chinese drug-dependent population, males exhibit higher levels of behavioral activation than females when faced with positive stimuli.

Overall, the revised Chinese version of the SPSRQ demonstrated suitability for use in drug-dependent populations, displaying good psychometric properties. Comprising 25 items measuring two factors – punishment sensitivity (SP; 14 items) and reward sensitivity (SR; 11 items) –the scale maintains the original SPSRQ's basic structure. The revised Chinese SPSRQ exhibited good item quality, internal structure validity, reliability, and convergent and discriminant validity evidence, making it a valid measurement tool to assess reinforcement sensitivity levels in Chinese drug-dependent populations. With some items removed, the revised version is also more time-efficient than the original and more suitable for large-scale surveys. Its specific application in future work includes assessing the extent of drug dependence, alcohol consumption and other forms of group dependence, and evaluating the outcomes of interventions.

Simultaneously, this study not only does expand the confirmed age range for the SPSRQ but also broadens its applicability to include person with substance use disorder. Future studies can test the revised SPSRQ further as a measurement tool in drug-addicted populations to enhance the precision of reinforcement sensitivity level assessments and increase the credibility of the findings. Additionally, it can be used to explore the mechanisms of reinforcement sensitivity behind substance addiction, informing the design of more individualized treatment programs according to the reinforcement sensitivity characteristics of subjects.

There are some limitations to this study. First, the drug treatment population is exclusively from compulsory isolation drug treatment centers, lacking representation of individuals in voluntary programs like community-based drug treatment programs. Although in this study, the scale was applied to the Chinese drug treatment population, its representativeness is somewhat limited to compulsory treatment. To expand the revised SPSRQ in drug rehabilitation populations, future studies should explore samples from voluntary programs, and expanding groups for further revision and refinement of the SPSRQ.

Secondly, the *CFI* and *TLI* values of the SPSRQ in this study did not meet the traditional criteria (>0.90). Based on the inconsistency of items across culturally various revised versions and the impact of numerous entries on *CFI* and *TLI*, future research should continue to develop an appropriate shortened version of the scale which would eliminate problematic entries performing poorly in various studies and samples. Alternatively, adjustments to specific item formulations in the original SPSRQ could be made, considering the study sample's characteristics and relevant theoretical rationale (O'Connor et al., 2004).

Finally, while reinforcement sensitivity is considered to be a relatively stable in person with substance use disorder, the cross-sectional design here does not rule out the possibility that the higher reward sensitivity in males could result from drug abuse. Follow-up studies should use a tracking design to allow for the possibility of drawing more definitive causal conclusions about the relationship between reinforcement sensitivity and drug addiction.

## Data availability statement

The raw data supporting the conclusions of this article will be made available by the authors, without undue reservation.

## Ethics statement

The studies involving humans were approved by Ethics Committee of the Second Xiangya Hospital of Central South University in China. The studies were conducted in accordance with the local legislation and institutional requirements. The participants provided their written informed consent to participate in this study.

## Author contributions

SL: Conceptualization, Formal analysis, Methodology, Writing – original draft, Writing – review & editing. DX: Conceptualization, Formal analysis, Methodology, Writing – original draft, Writing – review & editing. ML: Data curation, Validation, Writing – original draft. QC: Data curation, Validation, Writing – original draft. JZ: Funding acquisition, Project administration, Writing – original draft. XL: Conceptualization, Project administration, Supervision, Writing – review & editing. TC: Conceptualization, Project administration, Supervision, Writing – review & editing.
