# Supplementary material for: Exploratory and confirmatory factor analysis of the sensitivity to punishment and sensitivity to reward questionnaire-revised and its psychometric evaluation among Chinese person with substance use disorder
Source: Front Psychol. 2024 Jun 12;15:1351450. doi: 10.3389/fpsyg.2024.1351450 (PMC11199884; doi:10.3389/fpsyg.2024.1351450)
Supplement: Supplementary file 1 [file Data_Sheet_1.docx]

**Discussion**

The detailed process of deleting a specific project based on its semantic content is as follows:

In terms of the specific content of the items, as for sensitivity to punishment factor, item 1 (Do you often refrain from doing something because you are afraid of it being illegal?) reflects more on the individual's adherence to social norms; item 3 (Do you prefer not to ask for something when you are not sure you will obtain it?) tends to measure the individual's tolerance for uncertainty; item 11 (When you were a child, did you feel troubled by being punished?) may be affected by recall bias as it inquires about the subject's early experiences; and item 7 (Is it difficult for you to telephone someone you do not know?) , item 9 (Do you often give up your rights to avoid conflicts with others or organizations?) , item 17 (Are you a shy person?) , item 25 (Do you think a lot before complaining in a restaurant if your meal is not well prepared?) , item 27 (Would you be bothered if you had to return to a store when you noticed you were given the wrong change?) , and item 29 (Whenever you can, do you avoid going to unknown places?) , are related to specific social situations and may be more indicative of an individual's social willingness and ability, and thus the actual measures of these nine items have a weaker correlation with sensitivity to punishment.

And as for the sensitivity to reward factor, item 4 (Will you do something because you want to be valued by family or friends) may reflect the extent to which subjects seek approval externally; in items 8 (Do you like to take some drugs because of the pleasure you get from them?), 28 (Do you often have trouble resisting the temptation of doing forbidden things?) and 36 (Is it difficult to stop playing gambling games), keywords such as "drugs", "forbidden things " and " gambling " may seem sensitive to subjects in compulsory isolation drug rehabilitation centers, fearing potential negative consequences and therefore prone to falsification or falsification; items 32 (Is it easy for you to associate tastes and smells to very pleasant events?) and 34 (Are there a large number of objects or sensations that remind you of pleasant events?) both involve memories of happy events, which are related to specific personal experiences of the participants and do not have good universality; item 40 (Does your attention easily stray from your work in the presence of an attractive stranger?) focuses on reflecting attentional aspects, whereas items 2 (Does the good prospect of obtaining money motivate you strongly to do some things), 26 (Do you generally give preference to those activities that imply an immediate gain?), and 42 (Are you interested in money to the point of being able to do risky jobs?) are more oriented towards measuring impatient, impulsive personality traits, and thus these ten items are weakly associated with sensitivity to reward.

After sorting out the previous studies and the specific content of each item, it was found that the demerit of these 19 items were consistent across empirical studies in different cultures, and although they had a good degree of differentiation, there were different degrees of discrepancies with the original measurement objectives of the scale, and they could not accurately reflect the measurement content of the scale, so the final decision was made to delete the above mentioned 19 questionable items.
